# Frontal subcutaneous lipoma associated with large interhemispheric lipoma and corpus callosum agenesis

**DOI:** 10.1016/j.radcr.2021.12.016

**Published:** 2021-12-31

**Authors:** Dhara Rana, Sayali Kulkarni, Jamshed Zuberi, Fred Berlin

**Affiliations:** a Rowan School of Osteopathic Medicine, 1 Medical Center Dr, Stratford, NJ 08084, USA; b Department of Surgery, St. Joseph's University Medical Center, Paterson, NJ, USA; c Department of Interventional Radiology, St. Joseph's University Medical Center, Paterson, NJ, USA

**Keywords:** Interhemispheric intracranial lipoma, Neuroradiology, Seizures, Meninx, Subcutaneous lipoma

## Abstract

Intracranial lipomas are extremely rare fat-containing lesions that comprise 0.1%-0.5% of all primary brain tumors. They are congenital lesions that arise due to persistence and maldifferentiation of the meninx primitive (subarachnoid space precursor). We report a case of a 30-year-old woman who presented with seizures due to an intracranial lipoma and no neurological deficits. CT (computerized tomography) imaging findings demonstrated a large interhemispheric partially calcified lipoma that communicated with a large scalp lipoma and was associated with agenesis of the corpus callosum. Compared to the prior CT imaging, the lipoma increased in size from 3.4 cm to 4.1 cm transversely. A recent CT angiogram done due to suspicion of an aneurysm showed the lipoma now measuring 6 cm by 4.7 cm. Most cases of intracranial lipoma have been reported in the pediatric age group. Here, we report a rare case of interhemispheric intracranial lipoma in the adult age group. This case also demonstrates the importance of imaging modalities for detecting intracranial lipoma without performing invasive brain biopsy.

## Introduction

Intracranial lipomas are extremely rare fat-containing lesions that comprise 0.1%-0.5% of all primary brain tumors [1]. More than half of intracranial lipomas are associated with congenital malformation such as agenesis or dysgenesis of the corpus callosum [2]. These are usually detected incidentally or as a result of investigating clinical presentations of epilepsy or hydrocephalus. They are usually asymptomatic in presentation but can be accompanied by headaches, seizures, or symptoms due to mass effects. Several theories have been brought forward about the cause of intracranial lipomas. A favored theory is the persistence and maldifferentiation of meninx primitiva (subarachnoid space precursor). Brain computed tomography (CT) scan and brain magnetic resonance imaging (MRI) are 2 imaging modalities that can be used to make the diagnosis without a brain biopsy. Both imaging modalities can aid in identifying a lipomatous mass. Here, we present a rare case of a large interhemispheric intracranial lipoma with extension to the scalp in an adult woman.

## Case report

**Fig. 1:**
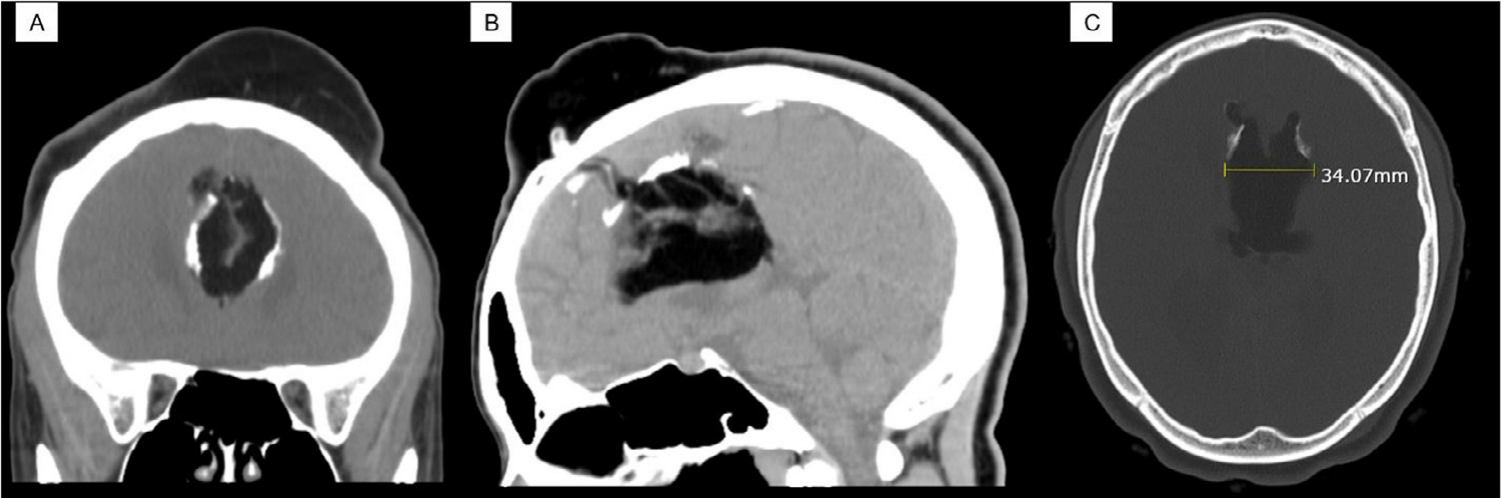
Head CT scan without contrast from November 2019. (A) Coronal view showing the interhemispheric lipoma. (B) Sagittal view showing the intracranial lipoma communicating with the scalp. (C) Axial view showing the interhemispheric and invasion into the body of the lateral ventricles bilaterally measuring about 3.4 cm.

A 30-year-old female patient presented with a history of seizures and headaches due to an intracranial lipoma which had worsened over the past few years. The patient had a significant family history of death due to a brain aneurysm on her maternal side. The patient was initially evaluated in the emergency department for seizures 2 years ago. At that time, a CT scan without contrast was done. It showed a large interhemispheric partially calcified lipoma, which appeared to extend superiorly and anteriorly communicating with a large scalp lipoma and 3 small defects in the left frontal lobe (Fig. 1B). Furthermore, the lipoma also extended into the body of the lateral ventricles bilaterally and agenesis of the corpus callosum was present (Figs. 1A and C). These findings were also seen in a prior CT head scan done 10 years ago. The patient's seizures were controlled with Levetiracetam (Keppra) 500 mg.

Last year, the patient was seen by a neurologist for her brain mass and management of her seizures. She was unable to describe her seizures. However, she was told by witnesses that her whole body shakes during the episodes. She also explained that she has an aura before her seizures which she described as “feeling funny,” Her seizures occur once or twice a month. The patient followed up with the neurologist regarding her seizures. The patient had experienced multiple seizures over the past year. Her first seizure resulted in dizziness and her whole body shaking which caused her to bite her tongue. On the physical exam, the patient had no neurological deficits and motor strength was 5/5 in all 4 extremities. Her sensations were intact to light touch and pinprick throughout the upper extremities and lower extremities. Reflexes 2+ and symmetric throughout, no dysmetria on a finger-to-nose exam, and gait was intact. Her Levetiracetam dosage was increased to 750 mg and she was referred to a neurosurgeon.

Later in the year, an electroencephalogram was performed which revealed a normal study. The patient had a follow-up with the neurologist to discuss her results. Here, the patient addressed difficulty tolerating the higher dose of Levetiracetam as it was causing headaches and panic attacks. Thus, she started to take half of the Levetiracetam and consequently continued to have breakthrough seizures. Her medication regimen was changed to Levetiracetam 500 mg in the morning and Levetiracetam 750 mg at night.

**Fig. 2:**
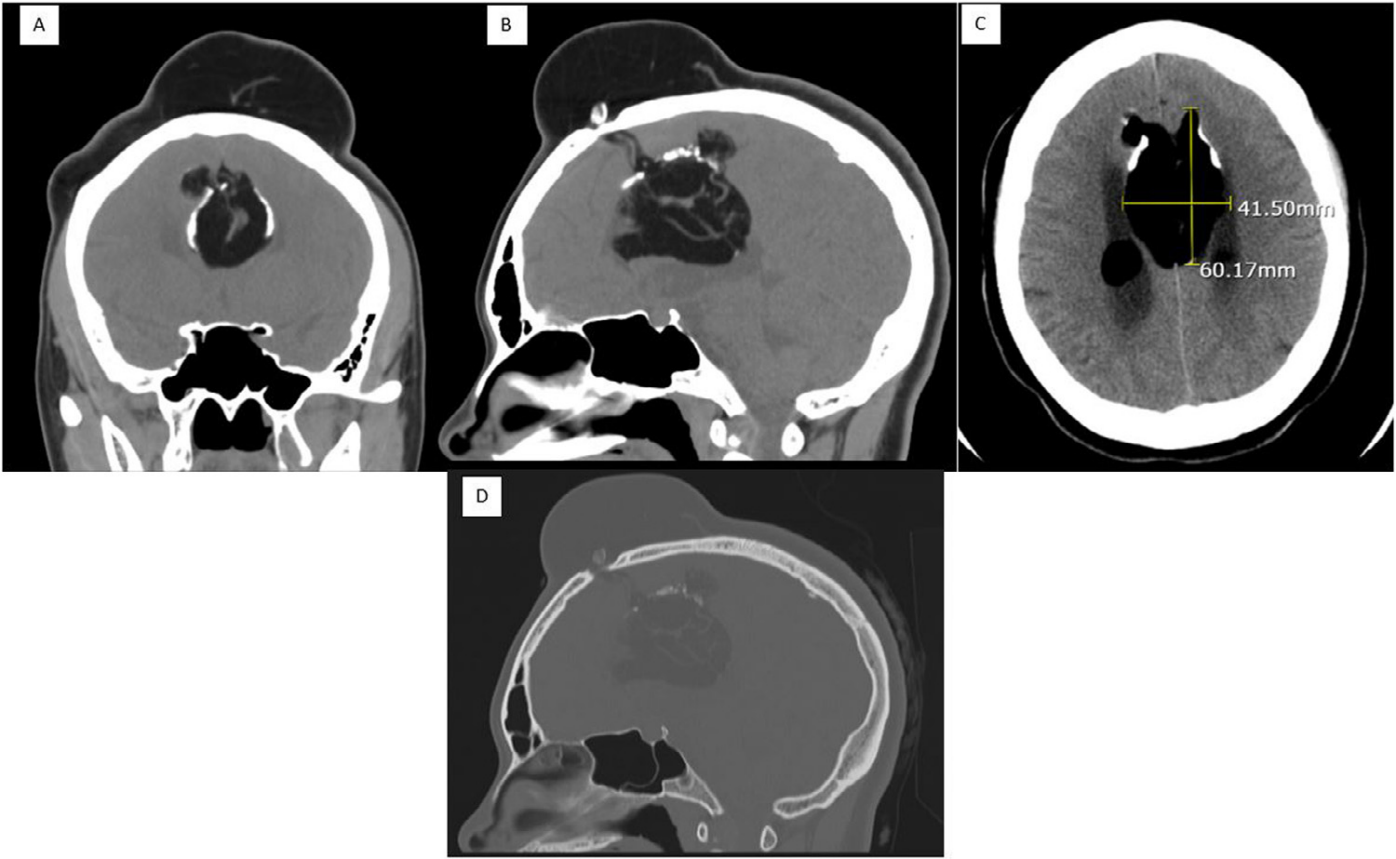
Head CT scan without contrast from May 2021. (A) Coronal view showing the interhemispheric lipoma. (B) Sagittal view showing the intracranial lipoma communicating with the scalp which has enlarged from the prior CT scan to 6 cm by 4.1 cm. (C) Axial view showing the interhemispheric and invasion into the body of the lateral ventricles bilaterally. (D) Sagittal view in bone window showing the intracranial lipoma communicating with the scalp.

**Fig. 3:**
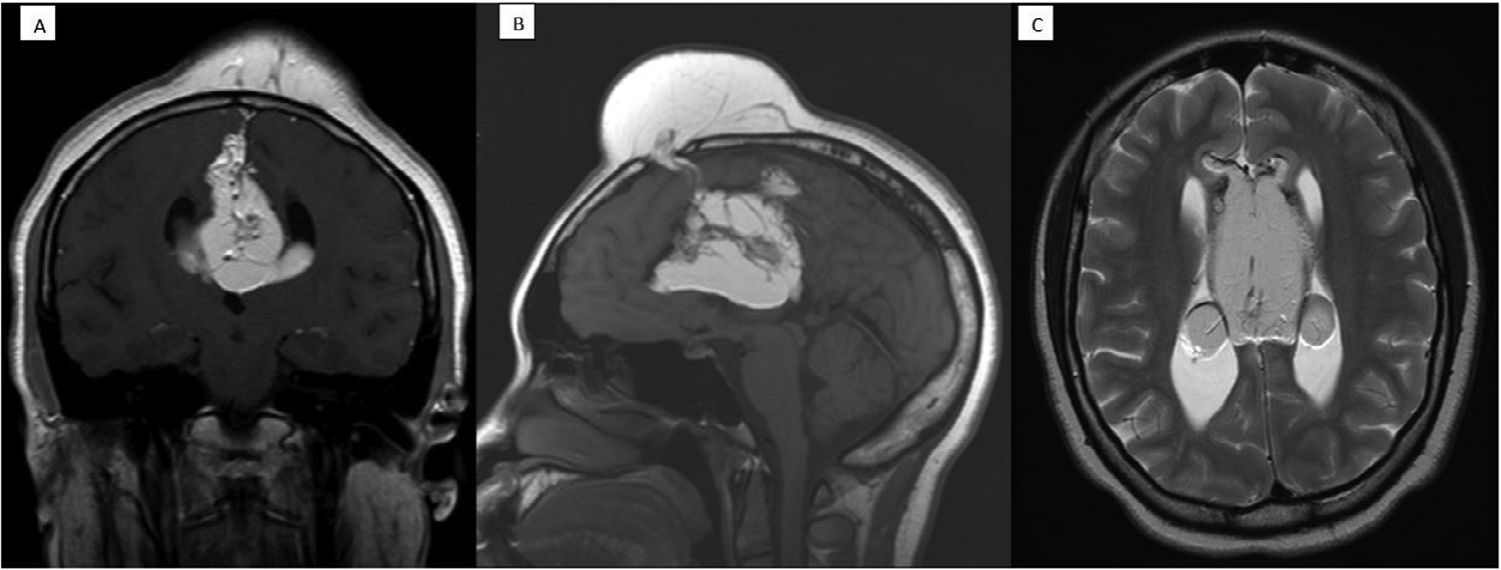
Head MRI from May 2021. (A) Coronal view showing the interhemispheric lipoma. (B) Sagittal view showing the intracranial lipoma communicating with the scalp. (C) Axial T2 view showing the interhemispheric and invasion into the body of the lateral ventricles bilaterally.

The patient underwent a brain MRI with and without contrast, and a head CT scan without contrast as requested by her neurosurgeon. The head CT showed a large interhemispheric partially calcified lipoma with superior and anterior extension and communication with the large scalp hematoma in the left frontal region with an interval increase in the size of the lipoma (Figs. 2A, B and C). In the current study, the lipoma measured about 4.1 cm in the transverse plane (Fig. 2C). Whereas in the prior study in this location the lipoma measured up to 3.4 cm transversely (Fig. 1C). There was a mild prominence of the ventricular system like the prior examination. The brain MRI demonstrated the large interhemispheric lipoma extending anterosuperiorly from the midline through the frontal bone and into the left and right frontal scalp soft tissues. This was shown to be larger on the current study as compared to the November 2019 CT study (Figs. 3A, B and C). Furthermore, the large interhemispheric lipoma with intraventricular extension was similar in size and appearance to the November 2019 CT study.

**Fig. 4:**
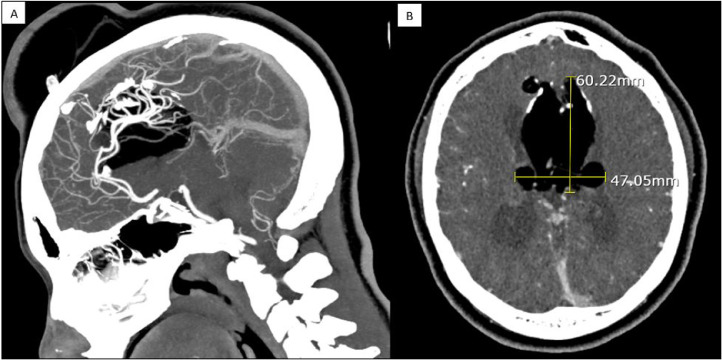
CT angiography of brain from June 2021. (A) Coronal view depicting blood vessel going through the lipoma. (B) Axial view show casing the measurement of the lipoma 6 cm by 4.7 cm.

Six months ago, the patient had a CT angiogram (CTA) done for suspicion of an unruptured cerebral aneurysm. The CTA revealed a large interhemispheric lipoma with agenesis of the corpus callosum communicating with the scalp. Parts of the callosal marginal branch of the anterior internal artery appeared to communicate with the diploic sinus. Most of the pericallosal artery and the internal cerebral veins runs right through the interhemispheric lipoma (Fig. 4A). The lipoma also extended within the lateral ventricles. In this study, the lipoma offically measured 6 cm by 4.7 cm (Fig. 4B). The posterior circulation was normal and no aneurysm was detected.

Oxcarbazepine (Trileptal) was added to her current regime of Levetiracetam 500 mg. Later in the year, the patient is planning to undergo neurosurgery for resection of the large interhemispheric lipoma.

## Discussion

Intracranial lipomas are congenital lesions because of abnormal differentiation of embryological meninx primitiva (subarachnoid space precursor). These lesions are a rare finding with an incidence of 0.08% and 0.46% in autopsy series [3]. In a review of 3000 brain CT scans of head trauma patients, only 3 lipomas were reported in 0.1% of the scans [3]. Although several theories tried to explain the cause of intracranial lipomas, one favorable theory is the concept of the meninx primitiva (subarachnoid space precursor) [4]. The proposed theory explained that an abnormal, persistent focus of the meninx primitive induced to differentiate into adipose and mature into lipoma because the meninx primitiva contains primitive perivascular reticuloendothelial which becomes specialized in the storage of fat [4]. Consequently, lipomas are not neoplastic but malformations. This idea also helped explain the subarachnoid, cisternal nature of the intracranial lipomas as well as intralesional location of vessels and nerves, and the absence of other mesodermal derivatives such as muscle [4]. Thus, the intracranial lipoma is maldifferentiated subarachnoid space and whatever courses through the cistern can course through the lipoma [4]. This is seen in our patient's CTA, where the pericallosal artery and internal cerebral vein coursed through the interhemispheric lipoma (Fig. 4A).

Most intracranial lipomas are asymptomatic and found incidentally. If symptomatic, patients commonly present with seizures, but can also present with raised intracranial pressure, dementia, and hemiparesis [5]. In a clinical study, the most frequent reasons for admission in 14 patients with intracranial lipoma were headache (50%), trauma (21.5%), epilepsy (21.5%), and symptoms due to mass effect (7%) [6]. Our patient presented with a history of seizures and headaches due to the intracranial lipoma and scalp swelling with no neurological deficit on physical exam.

Interhemispheric lipoma associated with a subcutaneous component is a rare finding. Most of the cases in the literature are associated in the pediatric population and only a few are found in adults [[7], [8], [9], [10], [11], [12]–13]. The most common type of intracranial lipoma is an interhemispheric lipoma which accounts for 45% of cases [14]. Other lesions are consist of quadrigeminal or superior cerebellar (25%), suprasellar/interpeduncular (14%), cerebellopontine angle (9%), and Sylvian fissure (5%) [14]. Our patient had a 6 cm by 4.7 cm interhemispheric partially calcified lipoma with a superoanterior extension, agenesis of the corpus callosum, and communication with the scalp in the left frontal lobe (Fig. 4B). The largest intracranial lipoma reported in the literature was by Durao et al of 60 × 35 mm, which is slightly smaller than the lipoma size that we presented [15].

Microscopically intracranial lipomas are composed typically of adipose tissue and a capsule with a variable quantity of collagen fibers, blood vessels, and calcifications [15]. Macroscopically, lipomas vary in size from subcentimeter to large masses and have a bright yellow, lobulated appearance. Calcifications may appear as curvilinear lines in nodular patterns in the periphery or center of a lipoma [16]. In our case, we observed calcification within the lipoma rather than in a curvilinear pattern and blood vessels going through the lipoma.

Both CT and MRI findings are characteristic of intracranial lipoma, so biopsy confirmation is not required for the diagnosis. Head CT scan is diagnostic as it shows fat density attenuation (-80 to -110 HU), calcifications, location of the lipoma, and associated anomalies [14]. Whereas MRI can be used to assess the anatomy of the corpus callosum, monitor the growth of the mass, and determine if any invasive characteristics [7,14]. Aside from that, MRI demonstrates short-T1 and T2 signals suggestive of fat as homogenous well-circumscribed lesions. In practice, if there is suspicion of intracranial lipoma, using fat density attenuation on a CT scan can help aid in determination. In practice, if there is a suspicion of intracranial lipoma, using the Hounsfield units (HU) from a CT scan can help confirm whether the mass is a lipoma, since the radiodensity measured would be like fat density attenuation (-80 to -110). Likewise, our patient showed an intracranial lipoma which communicated to the scalp. The CT scan revealed the intracranial mass with extremely low density ranging from -60 to -123 HU and the communicating extracranial mass also showed similar low radiodensity between -50 and -140 HU (Fig. 1B). With this, the intracranial and extracranial masses was diagnosed as a lipoma. A case study in literature reported extremely low densities of lipomas ranging between -96.5 and -125 similar to our findings [17].

Intracranial lipomas in most cases are asymptomatic and do not require treatment [5,6,14]. Otherwise, symptomatic intracranial lipomas are treated with medications, such as antiepileptic medications [6,14]. Our patient had seizures due to her intracranial lipoma, which were managed well with her anticonvulsant regime of Levetiracetam. Other options for treatment of intracranial lipomas are a surgical resection. Such treatment can be risky due to the adhesions of the collagenous capsule, and vessel and nerves surrounding the lipoma [6,7,14]. Microsurgical techniques can be an option via minimally invasive keyhole approaches which make it possible to remove the lipoma sparing risky structures such as blood vessels [7,18].

## Learning points

1. Intracranial lipomas are rare, asymptomatic, and incidental, which should be on a differential of a patient presenting with headaches or unexplained seizures.
2. CT and MRI are diagnostic for intracranial lipoma, so biopsy confirmation is not required as fat density and calcification can be detected on these imaging modalities.
3. Depending on size, location, vascularity involvement, and level of seizure, the patient may be potential surgical candidate.

## References

[1] Elhend SB, Belfquih H, Hammoune N (2019). Lipoma with agenesis of corpus callosum: 2 case reports and literature review. World Neurosurg.

[2] Alam A, Ram MS, Sahu S. (2006). Lipoma of the corpus callosum: diagnosis using magnetic resonance imaging. Medical J Armed Forces India.

[3] Gossner J. (2013). Small intracranial lipomas may be a frequent finding on computed tomography of the brain: a case series. Neuroradiol J.

[4] Truwit CL, Barkovich AJ. (1990). Pathogenesis of intracranial lipoma: an MR study in 42 patients. AJR Am J Roentgenol.

[5] Gastaut H, Regis H, Gastaut J (1980). Lipomas of the corpus callosum and epilepsy. Neurology.

[6] Yilmaz N, Unal O, Kiymaz N (2006). Intracranial lipomas—a clinical study. Clin Neurol Neurosurg.

[7] Aggarwal N, Gehlot KB, Kumar SD (2018). Frontal subcutaneous lipoma associated with interhemispheric lipoma, lipomeningocele, and corpus callosal dysgenesis in a young adult: CT and MRI findings. Indian J Radiol Imaging.

[8] Given CA, Fields TM, Pittman T. (2005). Interhemispheric lipoma connected to subcutaneous lipoma via lipomatous stalk. Pediatr Radiol.

[9] Karabağ H, Çakmak E, Çelik B (2014). Pericallosal lipoma associated with subcutaneous lipoma in an adult. J Neurosci Rural Pract.

[10] Karakas O, Karakas E, Boyacı FN (2013). Anterior interhemispheric calcified lipoma together with subcutaneous lipoma and agenesis of corpus callosum: a rare manifestation of midline craniofacial dysraphism. Jpn J Radiol.

[11] Kudoh H, Sakamoto K, Kobayashi N. (1984). Lipomas in the corpus callosum and the forehead, associated with a frontal bone defect. Surg Neurol.

[12] Mitilian D, Haddad D, Lenoir M (2009). Interhemispheric lipoma associated with frontal subcutaneous lipoma. J Plast Reconstr Aesthet Surg.

[13] Sarı A, Dinc H, Gümele H. (1998). Interhemispheric lipoma associated with subcutaneous lipoma. Eur Radiol.

[14] Yildiz H, Hakyemez B, Koroglu M (2006). Intracranial lipomas: importance of localization. Neuroradiology.

[15] Durão C, Pedrosa F. (2017). Undiagnosed intracranial lipoma associated with sudden death. Hum Pathol.

[16] Ayas ZÖ, Kotan D, Polat P. (2016). A rare case, diagnosed as calcified callosal lipoma, when the patient presented with acute stroke. Arch Med Sci Atheroscler Dis.

[17] Kazner E, Stochdorph O, Wende S (1980). Intracranial lipoma: diagnostic and therapeutic considerations. J Neurosurg.

[18] Chao S-C, Shen C-C, Cheng W-Y. (2008). Microsurgical removal of sylvian fissure lipoma with pterion keyhole approach—case report and review of the literature. Surg Neurol.

